# Comparison of Morphological and Genetic Characteristics of Avocados Grown in Tanzania

**DOI:** 10.3390/genes12010063

**Published:** 2021-01-04

**Authors:** Ibrahim Juma, Mulatu Geleta, Helena Persson Hovmalm, Agnes Nyomora, Ganapathi Varma Saripella, Anders S. Carlsson, Moneim Fatih, Rodomiro Ortiz

**Affiliations:** 1 Department of Plant Breeding, Swedish University of Agricultural Sciences, P.O. Box 101, 23053 Alnarp, Sweden; Mulatu.Geleta.Dida@slu.se (M.G.); Helena.Persson@slu.se (H.P.H.); ganapathi.varma.saripella@slu.se (G.V.S.); Anders.Carlsson@slu.se (A.S.C.); Moneim.Fatih@slu.se (M.F.); rodomiro.ortiz@slu.se (R.O.); 2 Department of Botany, University of Dar es Salaam, P.O. Box 35060, Dar es Salaam, Tanzania; agnesnyomora@gmail.com

**Keywords:** correlation, diversity, microsatellite, *Persea americana*, population structure

## Abstract

Tanzania has been growing avocado for decades. A wide variability of the avocado germplasm has been found, and the crop is largely contributing to the earnings of the farmers, traders, and the government, but its genetic diversity is scantly investigated. With the purpose of comparing morphological and genetic characteristics of this germplasm and uncovering the correlation between them and the geographical location, 226 adult seedling avocado trees were sampled in southwestern Tanzania. Their morphological characters were recorded, and their genetic diversity was evaluated based on 10 microsatellite loci. Discriminant analysis of principal components showed that the germplasm studied consisted of four genetic clusters that had an overall average gene diversity of 0.59 and 15.9% molecular variation among them. Most of the phenotypes were common in at least two clusters. The genetic clusters were also portrayed by multivariate analysis and hierarchical clustering for the molecular data but not for the morphology data. Using the Mantel test, a weak significant correlation was found between the genetic, morphological, and geographical distances, which indicates that the genetic variation present in the material is weakly reflected by the observed phenotypic variation and that both measures of variation varied slightly with the geographical sampling locations.

## 1. Introduction

Avocado (*Persea americana* Mill.) is an important fruit plant cultivated in tropical and subtropical climates. The fruit consumption is increasing worldwide, although much of the global production is in South and Mesoamerica [1,2]. *P. americana* is a polymorphic species with three botanical groups or horticultural races (the West Indian, Guatemalan, and Mexican) that are ecologically distinguishable [3]. Individuals in each botanical group also have some common genetic characteristics that distinguish them from members of other groups [4,5].

Evaluating the genetic variation existing in a given germplasm is essential to understand its potential application in crop breeding. The knowledge is also important in estimating the loss of genetic diversity, in providing proofs of the evolutionary forces shaping the genotypic variations, and also in selecting genotypes to be prioritized in conservation strategies [6]. Different markers have been used in avocado germplasm characterization, management, and conservation. Morphological markers were used to characterize avocado germplasm in California [7], Florida [8], Ghana [9,10], Mexico [11], Indonesia [12], and Tanzania [13], among others. However, besides being labor intensive, morphological traits are associated with some shortcomings, such as low variability (polymorphism) and heritability, late expression, influence by environmental factors, and subjectivity [14,15]. Nowadays, avocado germplasm characterization has been improved by the use of genetic markers, which can even discriminate closely related individuals. Some genetic markers that have been applied are isozymes [16], minisatellites [17], variable number tandem repeats (VNTRs) [18], randomly amplified polymorphic DNA (RAPD) [19], and restriction fragment length polymorphisms (RFLP) [20,21]. Others are inter-simple sequence repeats (ISSR) [22], simple sequence repeat (SSR) [15,23,24,25], and single nucleotide polymorphisms (SNPs) [5,26,27]. Choosing which marker type to employ in a diversity study depends on the study objectives and available financial resources, expertise, and facilities [28].

Population genetics has been used in describing the genetic composition of avocado populations and mechanisms affecting the composition [15,23,24,25]. Bayesian cluster analysis employed in the STRUCTURE and discriminant analysis of principal components has been widely utilized in studying the population structure of crops, including avocado [5,24,25,26,29]. Bayesian cluster analysis generates genetic clusters (genetic populations) with individuals in each cluster having distinctive allele frequencies at the investigated loci [30,31,32]. In avocado research, these genetic clusters have, sometimes, been shown to conform to the horticultural origin of the crop [4,5].

Tanzania rises from the sea level to more than 2900 m above sea level. The country has varying topographies, soils, and climates, which support the growth of different cultivars of avocados [13,25]. Although avocado is grown in several regions of Tanzania for the export and domestic markets [33], only two studies have been executed to characterize this germplasm based on morphological traits [13] and SSR markers [25]. The present study aimed to compare morphological and genetic characteristics of this germplasm and uncover correlations existing among the morphological and genetic characteristics and the geographical sampling locations. Such insights can provide important information for plant breeders to plan breeding programs in the future. In addition, the insights can increase awareness about avocado genetic resources that could be exploited for management and utilization in Tanzania.

## 2. Materials and Methods

### 2.1. Study Sites and Sampling

**Figure 1:**
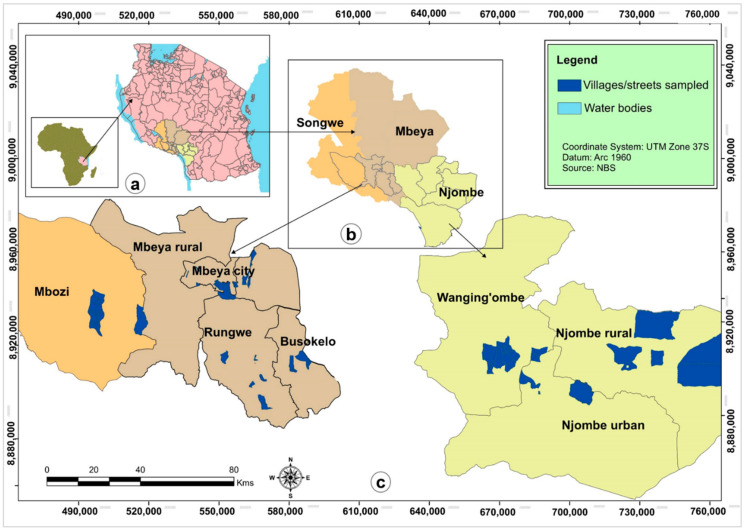
Study site map: (**a**) Top left is Tanzania’s location in Africa (small scale map) and the three avocado rich regions’ locations (large scale map); (**b**) Top center is the three regions showing the districts included in this research; (**c**) Bottom are the village/street locations in the districts.

The study sites were eight avocado-rich districts in the Mbeya, Songwe, and Njombe regions located in southwestern Tanzania (Figure 1). Two-hundred twenty-six seed originated adult avocado trees in 53 villages across the study sites were phenotyped during March through August 2017. Young leaf material of these trees was sampled, then dried and preserved using silica gel and later used for DNA extraction. The latitude and longitude of the collecting sites were determined with a Garmin Epix GPS mapping and multisport watch. The number of trees studied per district varied from 7 to 43 (Table 1).

### 2.2. Phenotyping

**Figure 2:**
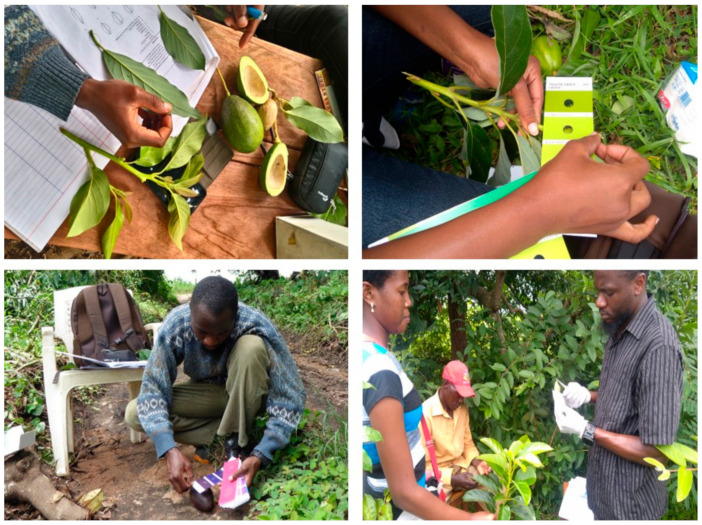
Measuring some morphological traits and collecting leaf samples.

Phenotypic characters of the 226 avocado trees were examined following the International Plant Genetic Resources Institute’s avocado crop descriptors [34]. Thirteen of the most important descriptors for avocado characterization were investigated. These descriptors included plant, fruit, and seed characteristics. The plant descriptors were the surface of the trunk, pubescence, and color of the young twig, the shape of and pubescence on the underside of the leaf, the number of primary leaf veins, and the leaf vein divergence at the middle of the leaf. The fruit descriptors included the shape of the mature fruit and pedicel, the peel thickness, and the flesh texture. For the seed, the descriptors assessed were the mature seed shape and its cotyledon surface. Color and peel thickness determination was achieved with the aid of the RHS color chart [35] and a ruler, respectively. Some phenotyping activities are presented in Figure 2.

### 2.3. DNA Extraction, Microsatellite Loci Amplification, and Genotyping

DNA was extracted from the dry avocado leaf tissue of the 226 trees using a Thermo Scientific genomic DNA purification kit following the protocol included in the kit. The analysis of the DNA integrity was done by running 1.2% agarose gel electrophoresis, whereas the DNA quality and quantity were checked with the NanoDrop spectrophotometer. Ten microsatellite loci of the sampled trees were investigated, of which nine were genomic, and one was an EST (expressed sequence tag) based microsatellite (Table 2). The ten microsatellite markers used were selected, based on their clear polymorphism pattern, from 16 highly polymorphic markers identified among 39 markers initially screened. The amplification of each locus was undertaken in 25 μL volume containing 25 ng genomic DNA, 0.3 μM of each of fluorescent-labeled forward primer and unlabeled reverse primer, 0.3 mM dNTPs, 1× PCR buffer, 1.5 mM MgCl_2_, and 1 U/μL *Taq* DNA polymerase. We used the S1000™ thermal cycler (BIO-RAD, Hercules, CA, USA) to run the PCR reactions under the program that involved initial denaturation at 94 °C for 60 s, followed by 35 cycles of denaturation at 94 °C for 60 s, primer annealing at primer-specific temperature for 30 s, and primer extension at 72 °C for 60 s. Then, the 35 cycles were followed with a final extension at 72 °C for 60 s. The capillary electrophoresis of the amplified products was carried out on the Applied Biosystems 3500 Genetic Analyzer (ThermoFisher Scientific, Waltham, MA, USA) using the GeneScan 500 LIZ size standard. The output, in the form of electropherograms, generated was imported to GeneMarker^®^ software V2.7 (SoftGenetics, State College, PA, USA) for visualization and allele-calling. The allele dataset at the 10 microsatellite loci was then organized in an Excel spreadsheet for further analyses.

### 2.4. Data Analysis

#### 2.4.1. Population Structure Analysis

We employed a discriminant analysis of principal components (DAPC) to infer the genetic clusters (subpopulations) and explore the population structure of the sampled trees using the allele dataset. The allele dataset in the GenAlEx format was first converted into a genind object using the R program df2genind [38], and then the DAPC was carried out on the genid object following the method described by Jombart and Collins [39]. The method involved the identification of the optimal number of genetic clusters (K) by using the find.clusters function and then employed the Bayesian Information Criterion (BIC) in choosing the optimal number of genetic clusters based on the elbow approach. Thereafter, the obtained clusters were further described by the DAPC. Since the genetic clusters derived from analysis of population structure might indicate the racial origin of avocado, all analyses of this work considered the genetic clusters as populations. This was also important for facilitating observation and comparison of the clustering of trees in the microsatellite and morphology-based multivariate analysis and hierarchical cluster analysis.

#### 2.4.2. Genetic Diversity among the Identified Clusters, Analysis of Molecular Variance (AMOVA) and Population Divergence

The total number of alleles scored, and the total number of different alleles observed were computed in HP-RARE [40]. Allelic richness (R_A_) and private allelic richness (R_PA_) were computed based on the rarefaction in HP-RARE. The estimation of the number of different alleles per locus, number of effective alleles, number of private, rare and common alleles per locus, Shannon’s information index, and average expected heterozygosity was done using GenAlEx 6.5 [41]. Average observed heterozygosity among the clusters was computed with Arlequin 3.5.2.2 [42]. The average gene diversity across the 10 loci for each cluster was computed with Arlequin. The global analysis of molecular variance (AMOVA) was performed on the clusters in Arlequin. Population divergence was assessed by comparing pairwise population F_ST_ and Nei’s genetic distance in Arlequin and GenAlEX, respectively.

#### 2.4.3. Genetic Relatedness among the Identified Clusters

Using GenAlEx, the Nei’s genetic distance was computed from the microsatellite data and then used for the principal coordinate analysis (PCoA) in the same software to study the relatedness of the trees with respect to their genetic clusters. The matrix used for PCoA consisted of 227 rows × 227 columns. The neighbor-joining dendrogram was computed in MEGAX [43] using Nei’s genetic distance matrix, and thereafter, the output in Newick-format was viewed and customized using the online tool iTOL v5 following Letunic and Bork [44].

#### 2.4.4. Phenotypic Characterization

Morphological characters of all the trees were organized in the Excel spreadsheet. Character variants that only occurred among some individuals of a particular cluster were identified. Principal components analysis of mixed data (PCAmix) [45] was performed on all morphological data to study morphological relatedness among the trees with respect to their genetic clusters (subpopulations). The analysis was carried out in XLSTAT version 2019.4.2 [46]. Thereafter, the dissimilarity matrix was computed in the same software from all morphological data. The matrix was used in producing a dendrogram to reveal morphological relatedness among the trees with regard to their genetic clusters. The dendrogram in the Newick format was produced in the R software using the Ward.D2 method [47,48]. The Newick format dendrogram was then viewed and customized using iTOL v5.

#### 2.4.5. Correlation between Genetic, Morphological, and Geographical Distances

The geographic distance matrix was computed from the latitude and longitude of the collecting sites in GenAlEx. Correlation between genetic, morphological, and geographical distance matrices was computed with the Mantel test at 999 permutations in the same software.

## 3. Results

### 3.1. Genetic Characterization

#### 3.1.1. Identification of Genetic Subpopulations (Clusters) and Description of Population Structure

**Figure 3:**
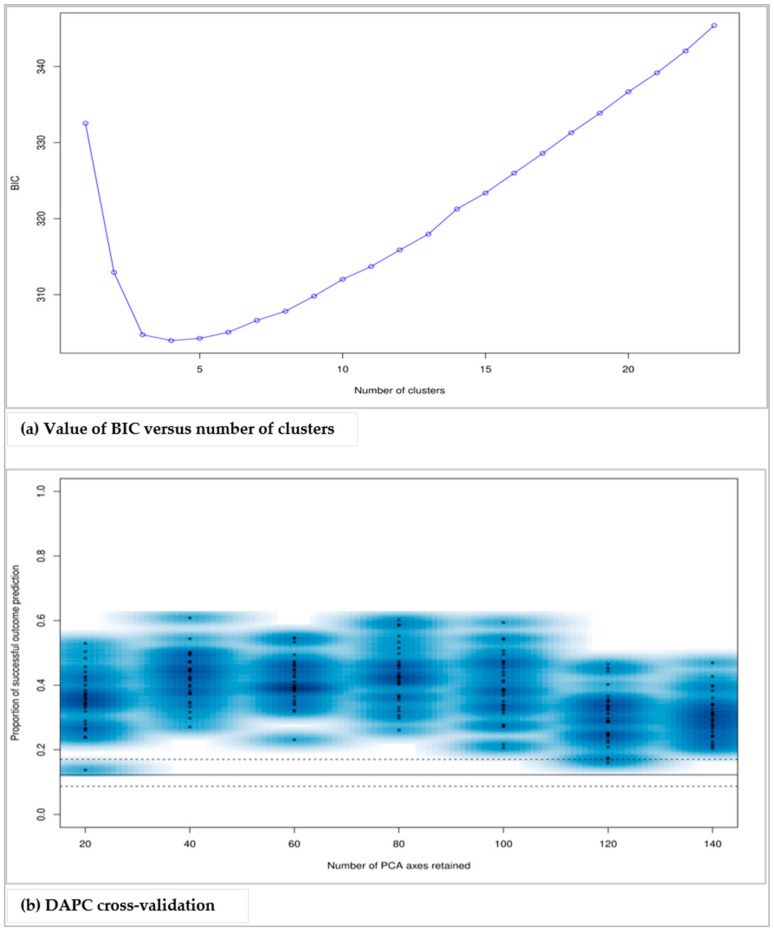
Determination of the optimum number of clusters (**a**) and number of principal components (PCs) and discriminant functions to be retained in the discriminant analysis of principal components (DAPC) analysis (**b**).

DAPC was employed to study the population structure of the sampled trees in detail. The ‘find.cluster’ function detected four clusters associated with the lowest BIC value (Figure 3a). These four clusters were considered to be useful in describing our data. Therefore, DAPC analysis was performed on the four clusters, and their proficient description was delivered. The forty-first PCs of the PCA, amounting to 81.2% of the total variance, and three discriminant functions were retained. These values were confirmed by a cross-validation analysis (Figure 3b).

**Figure 4:**
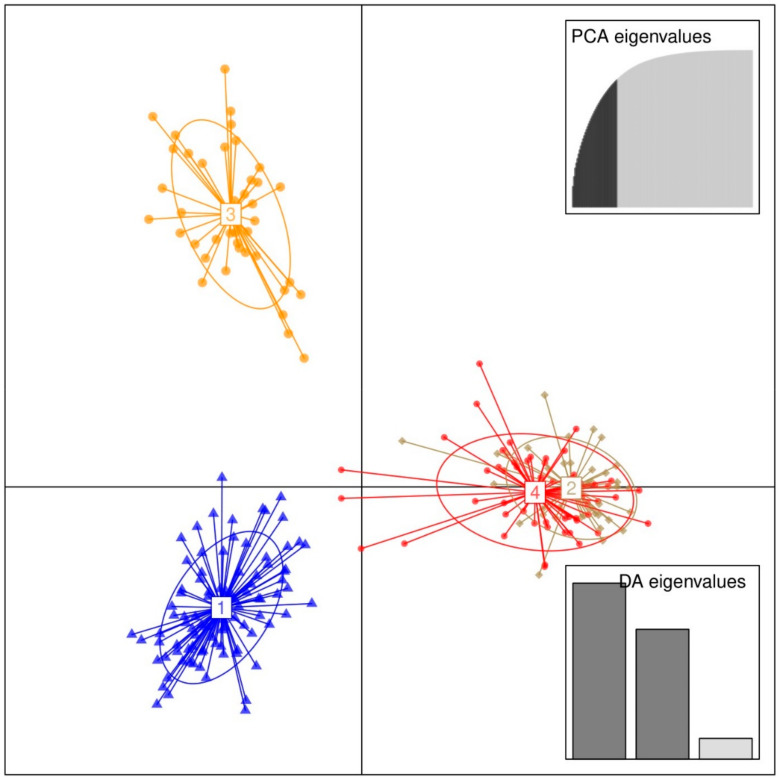
Discriminant analysis of principal components (DAPC) for 226 avocado samples. The axes represent the first two Linear Discriminants (LD). Each circle represents a cluster, and each symbol represents an individual. Numbers represent the different subpopulations identified by DAPC.

**Figure 5:**
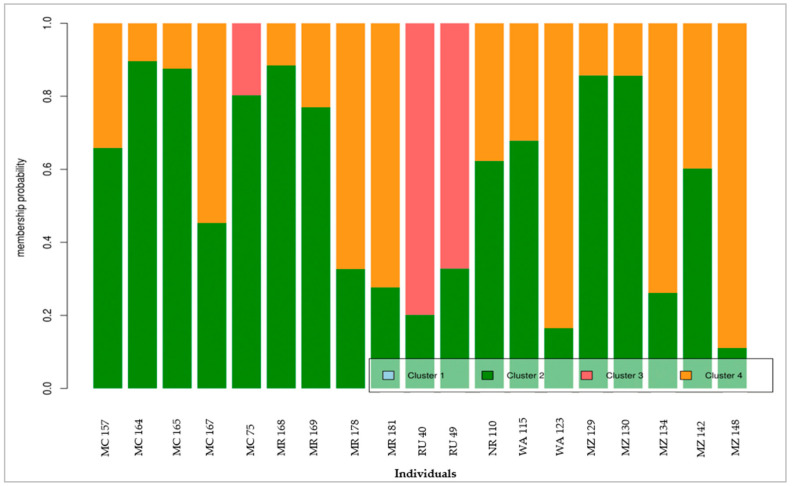
The distribution pattern of the alleles of different clusters for the most admixed individuals revealed by DAPC.

The DAPC plot (Figure 4) showed four clusters, with the linear discriminant 1 separating clusters 1 and 3 (to the left) from clusters 2 and 4 (to the right). The linear discriminant 2 only separated cluster 1 from cluster 3. Of the four clusters, cluster 1 was the largest with 90 individuals, followed by cluster 4 with 53 individual samples (Table 3). Cluster 2 and cluster 3 had a similar number of individuals, 42 and 41, respectively. In cluster 1, Rungwe had the highest number of individuals (32), followed by Busokelo (18). Neither the Mbeya city nor the Mbozi district contributed samples to this cluster. In cluster 2, the Njombe rural and Mbeya rural contributed a similar number of samples (12 and 11, respectively), whereas only two samples came from the Wanging’ombe district. No samples from Rungwe, Busokelo, or Njombe could be found in cluster 2. Cluster 3 had samples from two districts only, Mbeya city and Mbeya rural, which contributed 23 and 18 samples, respectively. Cluster 4 had more samples from the Mbozi district samples (17), followed by the Mbeya city (12), whereas only two samples came from Rungwe. The Njombe rural and Wanging’ombe districts had a similar contribution with 9 and 7 samples, respectively. Neither Busokelo nor Njombe urban contributed samples to this cluster. Further investigation of the individuals in each cluster revealed that the most ‘admixed’ individuals, i.e., individuals having a maximum of 90% probability to be a member of a single cluster, were nineteen among all samples (Figure 5). The allele composition of the four clusters for all the studied trees is presented in Figure S1.

#### 3.1.2. Genetic Diversity among the Four Genetic Clusters

The total number of alleles scored among the four clusters ranged from 727 (Cluster 3) to 1414 (Cluster 1), while the total number of different alleles observed ranged from 66 (Cluster 3) to 118 (Cluster 4; Table 4).

The analysis of allele frequency of the different clusters (populations) revealed that the mean number of different alleles per locus was lowest in cluster 3 (6.60) and highest in cluster 4 (11.80). The lowest and the highest private allele richness was recorded in cluster 3 (1.00) and cluster 1 (2.09), respectively. The effective number of alleles was lowest in cluster 3 (3.62) and highest in cluster 4 (5.68). Gene diversity, the unbiased expected and observed heterozygosity were lowest in cluster 1, i.e., 0.55, 0.70, and 0.60, respectively, pointing to a lower diversity among individuals of this group compared to other groups. The gene diversity was highest in cluster 3, i.e., 0.63, while allelic richness, unbiased expected heterozygosity, and the Shannon information index were highest for cluster 4, i.e., 9.48, 0.79, and 1.93, respectively, pointing to a higher diversity in these two avocado groups.

The number of alleles unique to a specific cluster, i.e., private alleles, per locus was lowest and highest in cluster 2 (0.5) and cluster 1 (2.3; Table 4). The least frequent private allele was an allele of 82 bp at the locus AVAG22, which had a frequency of 0.6% in cluster 1 (Table S1). The most frequent private allele was a 184 bp allele at the locus LMAV14, having a frequency of 61.5% in cluster 1. The number of alleles with a frequency of less than 5% in a population, i.e., rare alleles, per locus ranged from 1.8 (cluster 3) to 5.8 (cluster 4). The number of common alleles, with a frequency above or equal to 5% among the populations, per locus varied from 4.5 (cluster 1) to 6.0 (cluster 4). The most frequent common alleles were a 92 bp allele at the locus AVAG05, which had a frequency of 76.3% in cluster 1, followed by a 199 bp allele at the locus LMAV24, which had a frequency of 65.9% in cluster 2.

#### 3.1.3. Genetic Relationship among the Studied Avocado Samples

**Figure 6:**
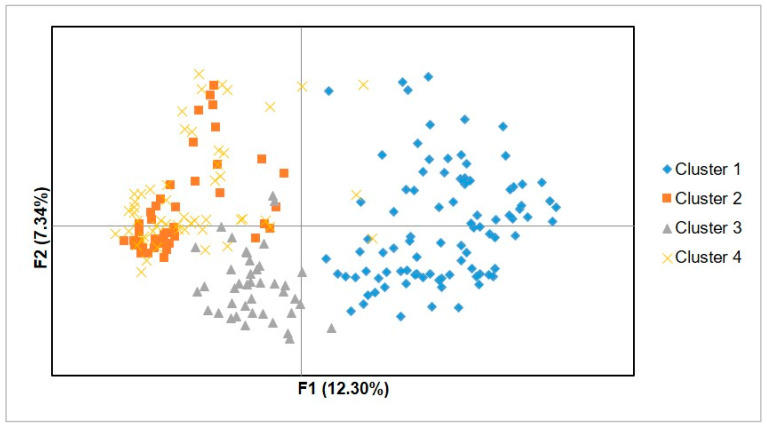
Principal coordinate analysis (PCoA) demonstrating the genetic relationships among individuals of the four clusters identified through DAPC.

PCoA was used to study the genetic relationship among the investigated avocado trees. The grouping pattern of the trees in the PCoA (Figure 6) was more or less similar to the DAPC findings, i.e., a grouping of samples into four clusters. The first two principal axes explained 19.64% of the total variation. Some individuals of cluster 2 and cluster 4 were projected on almost similar positions, while all individuals of cluster 1 and cluster 3 were resolved into distinct positions.

**Figure 7:**
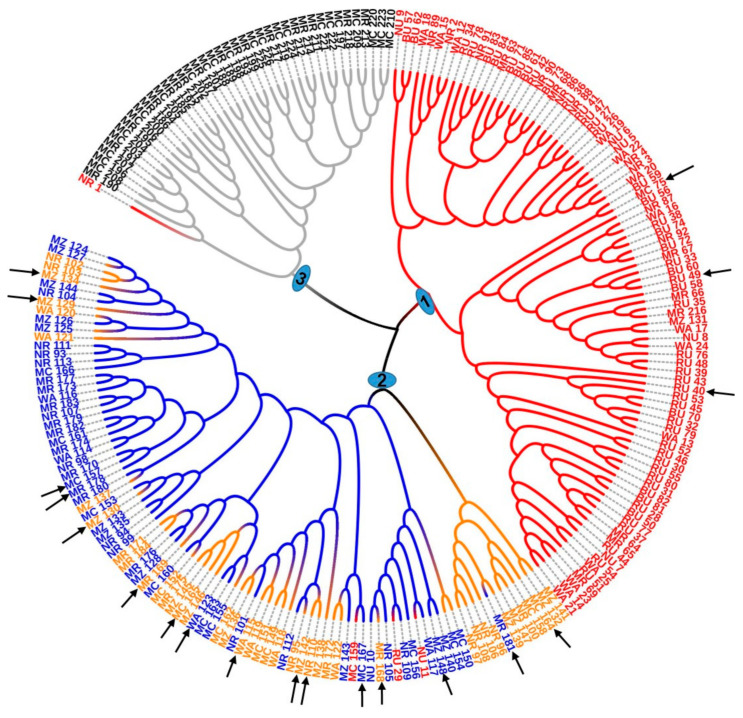
A simple sequence repeat (SSR) based dendrogram of the genetic relationship between the 226 avocado samples showing three major groups; group 1 and 3 correspond to clusters 1 and 3, respectively, and group 2 was a mosaic of individuals of two closely related clusters, i.e., cluster 2 (in orange) and cluster 4 (in blue) with three individuals from cluster 1 (in red). Samples marked with the same color belong to the same cluster. Highly admixed samples are indicated with arrows.

The Nei’s genetic distance matrix of the 226 avocado samples was used to study the genetic relationship among the four clusters identified by the DAPC. The dendrogram derived through the neighbor-joining cluster analysis method resolved clusters 1 and 3 into distinct groups except in a few cases (corresponding to group 1 and 3, respectively, in Figure 7). Group 2 contained samples from clusters 2 (in orange) and 4 (in blue), which suggests that members of the two clusters had higher genetic relatedness compared to the other clusters.

#### 3.1.4. Analysis of Molecular Variance and Population Differentiation

Analysis of molecular variance showed a higher molecular variance among the four avocado clusters (15.91%) than among individuals within clusters (9.91%), with the within individuals variance being the highest, 74.18% (Table 5).

The genetic differentiation among the four clusters identified by DAPC was investigated further by computing population pairwise F_ST_ (Table 6), and the analysis revealed significant differentiation among all pairs of clusters. The highest genetic differentiation was observed between clusters 1 and 2 (F_ST_ = 0.174), whereas clusters 2 and 4 displayed the lowest differentiation (F_ST_ = 0.062). Likewise, the analysis of the Nei’s genetic distances between clusters revealed the largest genetic distance between clusters 1 and 2 (1.163) and the lowest distance between clusters 2 and 4 (0.310). Cluster 1 had the largest mean F_ST_ (0.077) and genetic distance (0.735) from the other three clusters. The lowest mean F_ST_ (0.055) and genetic distance (0.0486) from the other three clusters were recorded in cluster 4 and 3, respectively.

### 3.2. Morphological Characterization 

#### 3.2.1. Morphological Characteristics among Individuals of the Genetic Clusters

Analysis of morphological characteristics among individuals of the four clusters revealed that the majority of the phenotypes appeared in at least two clusters (Table 7).

#### 3.2.2. Morphological Relationships among Individuals of the Four Clusters 

**Figure 8:**
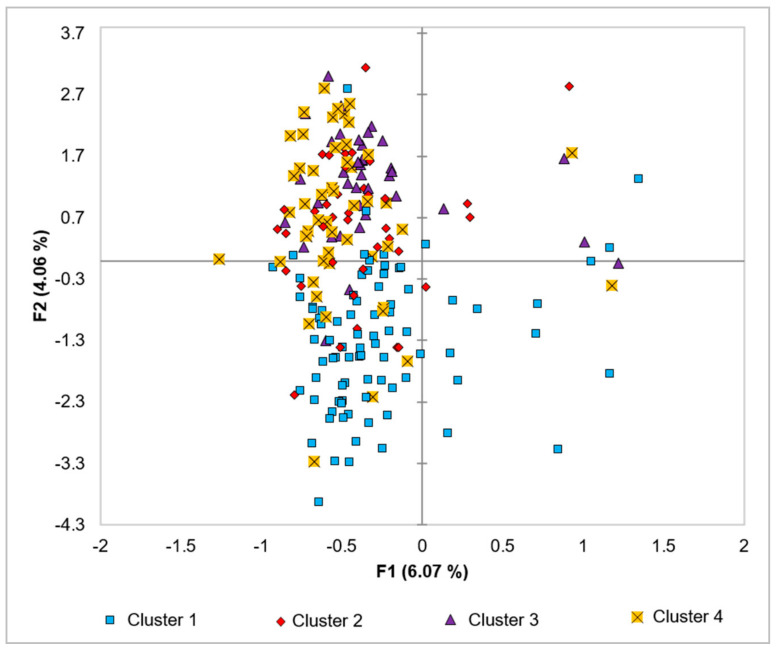
Principal components analysis of mixed data (PCAmix) demonstrating the morphological relationships among individuals of the four clusters.

**Figure 9:**
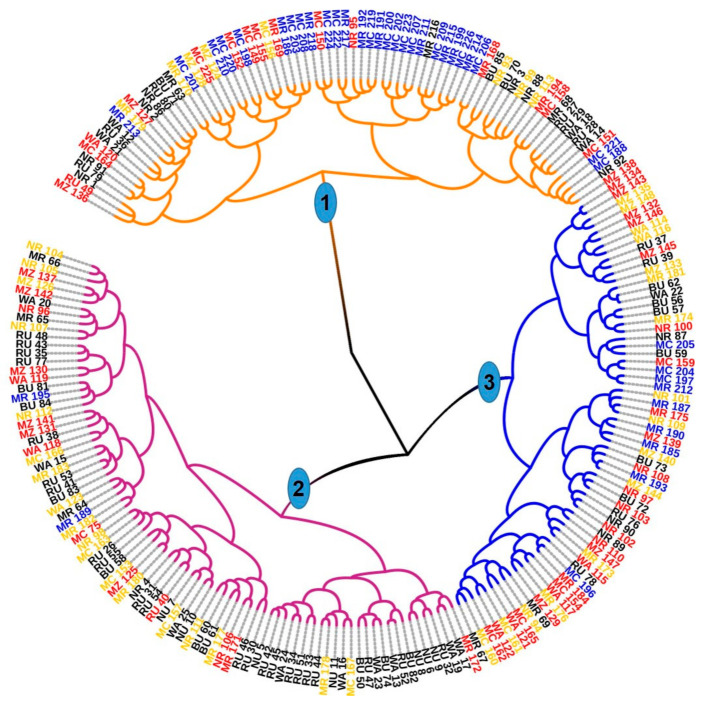
Morphological trait-based dendrogram of the 226 avocado trees, which grouped the trees into three major groups, with each group being composed of individuals from all the four genetic clusters. Note: samples marked with the same color belong to the same genetic cluster.

Morphology based-principal components analysis of mixed data (PCAmix) of the investigated trees showed intermingling of the individuals from the four genetic clusters with the first two axes showing a cumulative variation of 10.13% of the total variation (Figure 8). Similar results were noted in the morphology-based dendrogram in which the avocado trees were clustered into three groups, with each group containing individuals from all four clusters (Figure 9).

### 3.3. Correlation between Genetic, Morphological and Geographic Distances

The Mantel test indicated a low positive but statistically supported correlation between the genetic and geographical distances (*r* = 0.15, *p* = 0.001; Figure S2), between the morphological and geographical distances (*r* = 0.08, *p* = 0.001; Figure S3) and between the genetic and morphological distances (*r* = 0.11, *p* = 0.001; Figure S4) when the analysis was performed on individual samples.

## 4. Discussion

The present study has demonstrated the effectiveness of the genetic markers (microsatellite markers) over traditional morphological markers in characterizing avocado, exploring the diversity and the relationships among the individuals. Likewise, the study has shown the utility of DAPC in establishing the population structure of avocado crops and providing in-depth information on the individuals of the identified genetic clusters, which is an important step for practical plant breeding and conservation.

High diversity was noticed among the individuals of the four genetic clusters at the ten microsatellite loci. The mean number of different alleles per locus among the four clusters ranged from 6.60 (cluster 3) to 11.80 (cluster 4), with an average of 9.40 across the four clusters and loci (Table 4). Gross-German and Viruel [37] found a range of 3.7 (West Indian group) to 7.10 (hybrid group) with an average of 5.58 for the four populations they investigated, which consisted of a total of 41 avocado samples. Boza et al. [4] reported a range of 7.93 (Mexican group) to 9.78 (Guatemalan group), among the three horticultural groups, with a much higher overall mean of 9.09. Similarly, Schnell et al. [23] got a range of 6.00 (Mexican × West Indian group) to 13.35 (Mexican group) with an overall average of 10.26 for six populations of avocado comprising 221 samples. Cañas-Gutiérrez et al. [49] reported a lower overall mean, 4.46 for 18 geographical populations. In the present work, allele richness was lowest in cluster 3 (6.00) and highest in cluster 4 (9.48) with an overall mean value of 7.69. This suggests that clusters 3 and 4 were the least and the most genetically diverse clusters, respectively. The most genetically diverse groups would be offered protection in conservation programs, and they may provide the best plant materials for breeding programs, whereas the least genetically diverse groups would deserve special conservation management [50]. Guzmán et al. [24] recorded a comparatively lower allelic richness, 5.95 (Mexican group) to 6.22 (West Indian group) with an overall average of 6.10, for the three avocado racial groups. While the current study’s private allele richness ranged from 1.00 (cluster 3) to 2.09 (cluster 1) with an overall average of 1.45, Guzmán et al. [24] recorded a range of 0.63 (Mexican group) to 0.89 (Guatemalan group) with an overall mean of 0.74 for the three avocado populations. The average observed and expected heterozygosity for the four clusters was found to be 0.65 and 0.74, respectively. Lower values were reported by Boza et al. [4], *Ho*: 0.53 and *He*: 0.64, for the three horticultural races included in their study. Higher values were estimated by Gross-German and Viruel [37], *Ho*: 0.66 and *He*: 0.71 (four populations), and Schnell et al. [23], *Ho*: 0.71 and *He*: 0.77 (six populations), indicating a comparatively higher diversity. While the overall average gene diversity in the present work was 0.59, Boza et al. [4] obtained a higher value (0.63) for the three avocado races they investigated.

The number of private alleles per locus ranged from 0.50 (cluster 2) to 2.30 (cluster 1) with a grand mean of 1.23 across all populations and loci (Table 4). Private alleles are a measure of population differentiation, thus the highest value for the number of private alleles per locus detected in cluster 1 indicates the greatest genetic differentiation of this cluster as was also revealed by its largest mean F_ST_. Boza et al. [4] reported the number of private allele per locus ranging from 0.65 (Mexican group) to 0.71 (West Indian group) among the three avocado races, and 0.02 to 0.07 among their six hybrid groups with a grand mean value of 0.23 for the nine populations, which is lower than the value obtained in our study. While, in the present study, the lowest and highest number of rare alleles per locus was 1.80 (cluster 3) and 5.80 (cluster 4), Boza et al. [4] got a range of 3.31 (Mexican group) to 6.24 (West Indian group) among the three botanical groups, and 0.00 to 3.44 among their six hybrid groups. Rare alleles are significant in plant breeding as they may be associated with adaptations to biotic and abiotic stresses [51]. In our study, the number of common alleles per locus varied from 4.40 (cluster 1 and cluster 3) to 6.00 (cluster 4), whereas Boza et al. [4] got a range of 3.22 (West Indian group) to 4.67 (Guatemalan group) among the three botanical groups, and 3.80 to 4.64 among their six hybrid groups.

The PCoA (Figure 6) and dendrogram (Figure 7) obtained from microsatellite marker-based analyses resolved the studied trees into groups that were more or less similar to the four genetic clusters established by the DAPC analysis. Gross-German and Viruel [37] observed that the model-based (STRUCTURE) genetic clustering, PCoA, and cluster analysis results were in line with the distribution of avocado into botanical races, i.e., the Mexican, West Indian, and interracial Guatemalan × Mexican. Similarly, Alcaraz and Hormaza [15] observed that the UPGMA based dendrogram grouped 75 avocado accessions into three major groups that mainly corresponded to the botanical races. The four genetic clusters (groups) generated in the present study might represent the three avocado races and a hybrid group. This was also indicated by Juma et al. [13], as Tanzanian avocado germplasm analyzed using different morphological traits was shown to contain material from all three races. Traits included were trunk surface and peel thickness. Smooth trunk surface was reported as an attribute of the Mexican and Guatemalan races, and the rough and very rough trunk surface is attributed to the West Indian race [52]. Thin ripe peel (≤1 mm thick) is ascribed to the West Indian and Mexican races, and a thick ripe peel (2–3 mm thick) was ascribed to the Guatemalan race [53]. Other traits were the doughy and buttery flesh textures ascribed to the Guatemalan and Mexican races and the watery flesh texture attributed to the West Indian group [53]. However, in the present study, the examination of these characteristics showed that they appeared among individuals of all four clusters. More genetic studies need to be carried out on the Tanzanian avocado germplasm together with representative samples of the three avocado races to confirm the germplasm’s racial origin.

The AMOVA indicated that the overall genetic differentiation among the four avocado genetic clusters, F_ST_, was 0.159 (*p* < 0.0001). This implies a substantial amount of diversity harbored by the trees investigated and that the four genetic clusters were significantly distinct. The level of population differentiation (F_ST_) observed in this study was higher than the values reported by Juma et al. [25] for the same plant material when AMOVA was carried out on district-based populations (F_ST_ = 0.061, *p* < 0.0001) and altitudinal groups (F_ST_ = 0.025, *p* < 0.0001). Gross-German and Viruel [37] and Boza et al. [4] found an overall population differentiation of 0.25 and 0.193, respectively, which are comparatively higher than the value obtained in our study. In both studies, populations were based on the racial origin of avocado. Contrary to that, Cañas-Gutiérrez et al. [49] noted an overall population differentiation of 0.054 among the municipality-based populations, which is about 69% less than the value observed in the present study. Considering the AMOVA-based findings from the mentioned studies, it can be concluded that the overall population differentiation among avocado groups is higher if the grouping is based on racial origin than if it is based on geographical origin.

Pairwise comparison of population differentiation (F_ST_) and divergence (Nei’s genetic distance) revealed significant differentiation among all the clusters, with the lowest differentiation/genetic distance between clusters 2 and 4 (0.310; Table 6). The comparatively low Nei’s genetic distance between clusters 2 and 4 explains why the two clusters were less resolved from one another on the DAPC and microsatellite-based PCoA and dendrogram.

The morphology-based-PCAmix and dendrogram did not group the analyzed trees into their genetic clusters. The two analyses showed the intermingling of the individual trees from the four clusters. This finding suggests that the SSR loci investigated were not linked to the genes governing the investigated morphological traits. Another explanation is that the environment significantly influenced the phenotypes if linkage exists.

A weak positive correlation was revealed between the geographical distance of the sampling locations and the genetic distance (*r* = 0.15, *p* = 0.001) and between the geographical distance and the morphological dissimilarity matrix (*r* = 0.08, *p* = 0.001). Prohens et al. [54] observed a lack of correlation between geographical distance and AFLP-based genetic distance (*r* = 0.11, *p* < 0.10) in their study of 28 Spanish eggplant accessions (*Solanum melongena* L). Contrary to our study, they observed a comparatively higher correlation between the geographical and morphological distances (*r* = 0.25, *p* < 0.01). Sreekumar et al. [55] reported a highly significant correlation between geographical distance and AFLP-based genetic distance (*r* = 0.73, *p* = 0.009), whereas no correlation could be found between geographical distance and morphological trait-based distance (*r* = 0.44, *p* = 0.07) in their study of 60 breadfruit samples (*Artocarpus altilis*) in India. The weak correlation between geographical and genetic or morphological distances observed in the present study could be due to persistent movements and sharing of seeds between farmers of different areas [13,25,33]. In the present study, a weak positive correlation was also noticed between the genetic and morphological distances (*r* = 0.11, *p* = 0.001). This suggests that there was no strong association between the studied morphological traits and the 10 SSR loci investigated. It also suggests that the morphological trait variation cannot fully display the pattern of genetic diversity in avocado. Working with 62 Ethiopian maize accessions, Beyene et al. [28] noticed a moderate positive significant correlation between AFLP-based genetic and morphological distances (*r* = 0.39, *p* = 0.001), and also between SSR-based genetic and morphological distances (*r* = 0.43, *p* = 0.001). In a similar study on Vietnamese and Cambodian sesame accessions, Pham et al. [56] reported a highly significant positive correlation (*r* = 0.88, *p* = 0.001) between agro-morphological and RAPD marker based distances between the accessions. Contrary to that, Roldan-Ruiz et al. [57] observed an absence of correlation between AFLP-based genetic and morphological distances (*r* = −0.06, *p* < 0.375) and a weak correlation between the sequence tag sites (STS)-based genetic and morphological distances (*r* = 0.18, *p* < 0.12) in 16 ryegrass varieties. Similarly, Sreekumar et al. [55] reported an absence of correlation between the AFLP-based genetic distance and the morphological distance (*r* = 0.01, *p* = 0.5) of breadfruit in India. Smith and Smith [14] asserted that phenotypic variation sometimes does not follow genetic variation due to the influence of the environment on the phenotypic expression of the genotypes and potential multiple gene action on the traits.

## 5. Conclusions

The findings from this study showed that the population structure of the analyzed avocado trees comprised four genetic clusters that might represent the racial origin of the germplasm: Mexico, Guatemala, and West India. Although the four clusters were genetically distinguishable, their morphological characters, even for the characters that were supposed to be found only in a particular avocado horticultural race (a cluster), were overlapping. The weak positive correlation observed between geographical and genetic or morphological distances indicates that the genetic and morphological characteristics of the studied trees varied slightly with the geographical locations. Similarly, the weak positive correlation observed between the genetic and morphological distances indicates a low level of agreement between the diversity patterns derived from the two distances.

## Figures and Tables

**Table 1:** Sampling information.

Region	District	Village/Street	Number of Trees Sampled per Village/Street	Number of Trees Sampled per District	Latitude and Longitude of the Collecting Sites
Mbeya	Mbeya city	ARI-Uyole	1	43	8°54′ S, 33°30′ E–9°26′ S, 33°23′ E
		Ituha	8		
		Tonya	6		
		Shewa	5		
		Mwahala	4		
		Isoso	7		
		Nduguye	6		
		Reli	6		
	Mbeya rural	Iyelanyala	3	43	8°51′ S, 33°36′ E–9°27′ S, 33°24′ E
		Idunda	3		
		Inyala	1		
		Nsongwi Juu	10		
		Nsongwi Mantanji	9		
		Ifiga	8		
		Hatwelo	1		
		Nzenga	8		
	Rungwe	Ikama	2	34	9°16′ S, 33°46′ E–9°35′ S, 33°40′ E
		Nkunga	2		
		Mahenge	1		
		Mibura	5		
		Mpuga	1		
		Katumba	3		
		Katusyo	4		
		Ntandabara	6		
		Ndembo	5		
		Katundulu	5		
	Busokelo	Ikambako	1	18	9°15′ S, 33°49′ E–9°34′ S, 33°45′ E
		Lukasi	7		
		Mbigili	5		
		Mbambo	5		
Njombe	Njombe urban	Ramadhani	3	7	9°19′ S, 34°42′ E–9°40′ S, 34°16′ E
		Buguruni	2		
		Ichuniro	1		
		Itulike	1		
	Njombe rural	Image	1	32	9°04′ S, 35°12′ E–9°40′ S, 34°16′ E
		Kahimbi	1		
		Mfriga	1		
		Madeke	1		
		Image	4		
		Matembwe	5		
		Isoliwaya	6		
		Kanuikelele	3		
		Ikondo	7		
		Mfriga	3		
	Wanging’ombe	Igima	4	24	9°11′ S, 34°32′ E–9°40′ S, 34°16′ E
		Kinenuro	5		
		Igodivaha	5		
		Imalinyi	5		
		Ilulu	5		
Songwe	Mbozi	Mahenje	11	25	9°17′ S, 32°49′ E–9°19′ S, 32°54′ E
		Igunda	6		
		Ndolezi	5		
		Shaji	3		

**Table 2:** The repeat motif of the simple sequence repeat (SSR) loci used in this study.

Locus Name *	Repeats
*AVAG05* ^1^	(AG)_10_
*AVAG22* ^1^	(GA)_15_
*AVMIX01* ^1^	(AT)_7_(AG)_12_
*ESTAVGA03* ^2^	(TC)_20_
*LMAV02* ^2^	(AC)_8_(AG)_14_
*LMAV14* ^2^	(AGAGGG)_4_(AG)_3_
*LMAV24* ^2^	(AG)_15_
*LMAV29* ^2^	(CTT)_8_(CT)_11_
*LMAV31* ^2^	(GA)_21_
*LMAV35* ^2^	(GAA)_5_(GA)_14_

^1^ = from Sharon et al. [36]; ^2^ = from Gross-German and Viruel [37]; * = all are genomic-SSRs except *ESTAVGA03*, which is an EST-SSR.

**Table 3:** Information on the number of samples in each cluster.

Site	Cluster 1	Cluster 2	Cluster 3	Cluster 4
Mbeya city	0	8	23	12
Mbeya rural	8	11	18	6
Rungwe	32	0	0	2
Busokelo	18	0	0	0
Njombe urban	7	0	0	0
Njombe rural	11	12	0	9
Wanging’ombe	14	3	0	7
Mbozi	0	8	0	17
Total	90	42	41	53

**Table 4:** Estimates of different genetic diversity parameters within the four genetic clusters.

Diversity Measurement	Cluster 1	Cluster 2	Cluster 3	Cluster 4	Grand Mean
*n*	90	42	41	53	NA
*A*	1414	750	727	883	NA
*A_O_*	97	95	66	118	NA
*Na*	9.70	9.50	6.60	11.80	9.40
*Ne*	3.85	4.69	3.62	5.68	4.46
*R_A_*	7.20	8.08	6.00	9.48	7.69
*R_PA_*	2.09	1.15	1.00	1.55	1.45
*Ho*	0.60	0.66	0.69	0.65	0.65
*He*	0.70	0.77	0.71	0.79	0.74
Gene diversity	0.55	0.62	0.63	0.56	0.59
*I*	1.58	1.74	1.44	1.93	1.68
*N_PA_*	2.30	0.50	0.80	1.30	1.23
*N_RA_*	5.20	4.70	1.80	5.80	4.38
*N_CA_*	4.50	4.80	4.80	6.00	5.03

*n*: Number of individuals, *A*: Total number of alleles scored, *A_O_*: Total number of different alleles observed *Na*: Number of different alleles per locus, *Ne*: Effective number of alleles, *N_RA_*: Number of rare alleles per locus, *N_PA_*: Number of private alleles per locus, *N_CA_*: Number of common alleles per locus, *R_A_*: Allelic richness, *R_PA_*: Private allelic richness, *Ho*: Observed heterozygosity, *He*: Expected heterozygosity, *I*: Shannon’s information index.

**Table 5:** Analysis of molecular variance (AMOVA) was conducted by grouping trees into their respective genetic clusters.

Source of Variation	Sum of Squares	Variance Component	Percentage Variation
Among clusters	200.47	0.69 Va	15.91
Among individuals within clusters	755.86	0.44 Vb	9.91
Within individuals	620.00	3.26 Vc	74.18
Total	1576.33	4.39	

Fixation indices and *p*-values: F_ST_: 0.159 (*p* (Va and F_ST_) < 0.0001); F_IS_: 0.118 (*p* (Vb and F_IS_) < 0.0001); F_IT_: 0.258 (*p* (Vc and F_IT_) < 0.0001).

**Table 6:** Pairwise differentiation of clusters (F_ST_) (above diagonal) and Nei’s genetic distance between clusters (below diagonal) and mean F_ST_ and genetic distance of each cluster from the other three clusters. All pairwise F_ST_ values were significant at *p* < 0.001.

	Cluster 1	Cluster 2	Cluster 3	Cluster 4	Mean F_ST_	Mean GD
Cluster 1	0.000	0.174	0.081	0.167	0.077	0.735
Cluster 2	1.163	0.000	0.139	0.062	0.058	0.588
Cluster 3	0.616	0.866	0.000	0.129	0.057	0.486
Cluster 4	1.158	0.310	0.850	0.000	0.055	0.595

GD = genetic distance.

**Table 7:** Frequency distribution of different phenotypic characteristics across the four clusters (cluster-specific phenotypes in bold).

Trait	Phenotype	Cluster 1	Cluster 2	Cluster 3	Cluster 4
Trunk surface	Smooth	0.053	0.044	0.080	0.066
	Rough	0.159	0.066	0.062	0.080
	Very rough	0.181	0.075	0.040	0.088
Twig surface	Glabrous	0.283	0.111	0.031	0.128
	Pubescent	0.111	0.075	0.150	0.102
Twig color	Yellow–green group 144	0.119	0.053	0.137	0.066
	Yellow–green group N144	0.088	0.022	0.004	0.009
	Speckled	0.049	0.071	0.035	0.111
	Green group 143	0.000	0.013	0.000	0.031
	Yellow–green group 145	0.027	0.013	0.000	0.009
	Yellow–green group 152	0.031	0.004	0.004	0.004
	**Yellow–green group 146**	**0.040**	0.000	0.000	0.000
	Yellow–green group 151	0.013	0.004	0.000	0.004
	**Yellow–green group 150**	**0.013**	0.000	0.000	0.000
	Others *	0.013	0.004	0.000	0.000
Leaf shape	Ovate	0.000	0.004	0.000	0.004
	Narrowly obovate	0.018	0.018	0.027	0.031
	Obovate	0.000	0.004	0.004	0.022
	Oval	0.274	0.053	0.102	0.088
	Roundish	0.058	0.027	0.009	0.013
	Lanceolate	0.040	0.049	0.027	0.040
	Oblong-lanceolate	0.000	0.022	0.004	0.022
	Other *	0.004	0.009	0.009	0.013
Leaf pubescence	Absent	0.049	0.062	0.040	0.097
	Sparse	0.128	0.013	0.004	0.013
	Intermediate	0.040	0.018	0.004	0.004
	Dense	0.177	0.093	0.133	0.115
Number of primary leaf veins	**11**	0.000	0.000	**0.004**	0.000
	**12**	**0.004**	0.000	0.000	0.000
	**13**	**0.009**	0.000	0.000	0.000
	14	0.022	0.013	0.000	0.004
	**15**	**0.018**	0.000	0.000	0.000
	16	0.071	0.000	0.013	0.009
	17	0.049	0.022	0.000	0.013
	18	0.058	0.040	0.040	0.044
	19	0.058	0.013	0.009	0.035
	20	0.053	0.035	0.053	0.066
	21	0.018	0.013	0.013	0.018
	22	0.013	0.035	0.022	0.018
	23	0.013	0.004	0.013	0.013
	24	0.000	0.004	0.004	0.009
	**25**	0.000	**0.004**	0.000	0.000
	26	0.004	0.000	0.004	0.004
	**29**	**0.004**	0.000	0.000	0.000
	**31**	**0.004**	0.000	0.000	0.000
	**33**	0.000	0.000	**0.004**	0.000
Primary leaf vein divergence	**34°**	0.000	0.000	0.000	**0.004**
	38°	0.009	0.009	0.000	0.000
	39°	0.004	0.004	0.004	0.000
	**40°**	**0.004**	0.000	0.000	0.000
	**41°**	**0.027**	0.000	0.000	0.000
	42°	0.004	0.000	0.004	0.009
	43°	0.004	0.004	0.004	0.009
	44°	0.018	0.013	0.022	0.013
	45°	0.004	0.013	0.004	0.013
	46°	0.031	0.004	0.009	0.004
	47°	0.031	0.022	0.013	0.004
	48°	0.018	0.018	0.009	0.022
	49°	0.022	0.013	0.018	0.018
	50°	0.035	0.022	0.013	0.031
	51°	0.040	0.009	0.022	0.022
	52°	0.022	0.018	0.018	0.000
	53°	0.013	0.009	0.022	0.013
	54°	0.027	0.004	0.000	0.018
	55°	0.009	0.009	0.009	0.004
	56°	0.009	0.000	0.004	0.027
	57°	0.013	0.000	0.000	0.004
	**58°**	**0.009**	0.000	0.000	0.000
	59°	0.004	0.009	0.000	0.004
	**61°**	**0.004**	0.000	0.000	0.000
	**62°**	**0.004**	0.000	0.000	0.000
	**68°**	**0.004**	0.000	0.000	0.000
	**69°**	**0.004**	0.000	0.000	0.000
Fruit shape	Oblate	0.035	0.009	0.004	0.004
	Spheroid	0.027	0.004	0.009	0.013
	High spheroid	0.027	0.009	0.004	0.013
	Ellipsoid	0.035	0.031	0.018	0.031
	Narrowly obovate	0.058	0.018	0.013	0.013
	Obovate	0.013	0.004	0.009	0.013
	Pyriform	0.049	0.027	0.018	0.031
	Clavate	0.009	0.022	0.000	0.018
	Rhomboidal	0.111	0.022	0.013	0.040
	Other *	0.018	0.035	0.088	0.058
Pedicel shape	Cylindrical	0.053	0.053	0.066	0.058
	Conical	0.257	0.124	0.088	0.146
	Rounded	0.053	0.000	0.009	0.027
	Inverted conical *	0.013	0.000	0.004	0.000
	Triangular prism *	**0.004**	0.000	0.000	0.000
	Biconcave *	0.000	**0.004**	0.000	0.000
Fruit skin thickness	≤1 mm	0.080	0.062	0.018	0.044
	2 mm	0.035	0.013	0.018	0.018
	**3 mm**	0.000	0.000	0.000	**0.004**
Flesh texture	Buttery	0.239	0.084	0.075	0.146
	Pastose (doughy)	0.027	0.035	0.080	0.031
	Granular	0.004	0.000	0.000	0.004
	Watery	0.080	0.044	0.062	0.018
Seed shape	**Oblate**	0.000	0.000	0.000	**0.004**
	Spheroid	0.013	0.000	0.000	0.009
	**Ellipsoid**	0.000	**0.004**	0.000	0.000
	Ovate	0.009	0.013	0.009	0.013
	Broadly ovate	0.044	0.044	0.066	0.058
	Cordiform	0.040	0.018	0.009	0.027
	Base flattened, apex rounded	0.150	0.044	0.053	0.049
	Base flattened, apex conical	0.124	0.049	0.031	0.058
	Other *	0.000	0.009	0.009	0.013
Cotyledon surface	Smooth	0.133	0.049	0.049	0.084
	Intermediate	0.088	0.084	0.058	0.066
	Rough	0.164	0.053	0.071	0.080

* = Figures describing the leaf, fruit, pedicel, and seed shapes, and description of the phenotypes grouped in the ”Other” category are available in Juma et al. [13].

## Supplementary Materials

The following are available online at https://www.mdpi.com/2073-4425/12/1/63/s1, Figure S1: The distribution pattern of the alleles of different clusters for all avocado trees, Figure S2: Mantel test showing the correlation between genetic and geographical distances, Figure S3: Mantel test showing a correlation between dissimilarity matrix and geographical distance, Figure S4: Mantel test showing a correlation between dissimilarity matrix and genetic distance, Table S1: Allele frequencies by locus for the four genetic clusters.
