# Supplementary figures and images for: Comparison of Morphological and Genetic Characteristics of Avocados Grown in Tanzania
Source: Genes (Basel). 2021 Jan 4;12(1):63. doi: 10.3390/genes12010063 (PMC7824774; doi:10.3390/genes12010063)

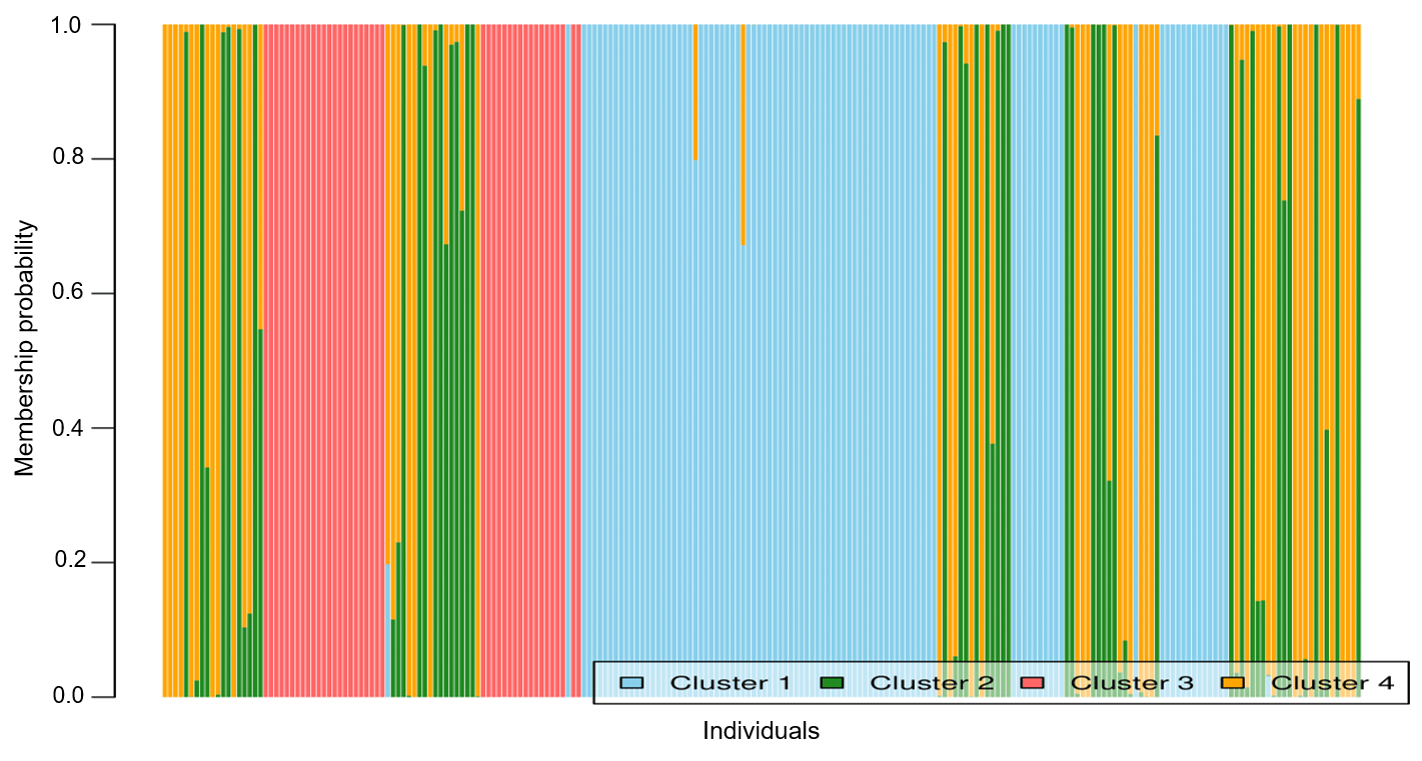

Supplement: Supplementary file 1 [file genes-12-00063-s001.zip › Supplementary files/Figuse S1.png]

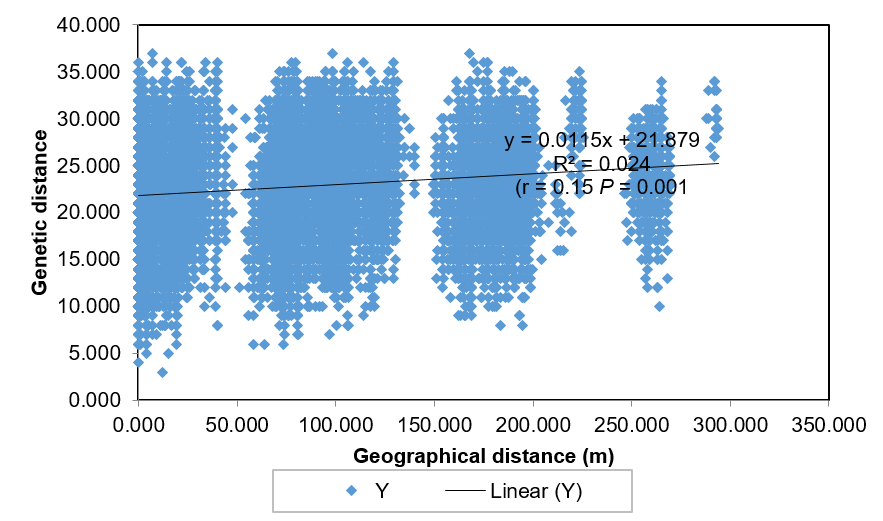

Supplement: Supplementary file 1 [file genes-12-00063-s001.zip › Supplementary files/Figuse S2.png]

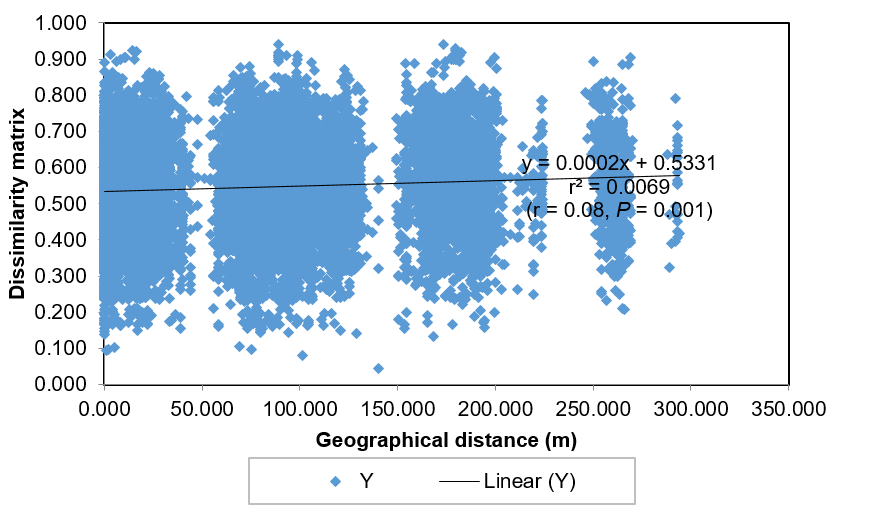

Supplement: Supplementary file 1 [file genes-12-00063-s001.zip › Supplementary files/Figuse S3.png]

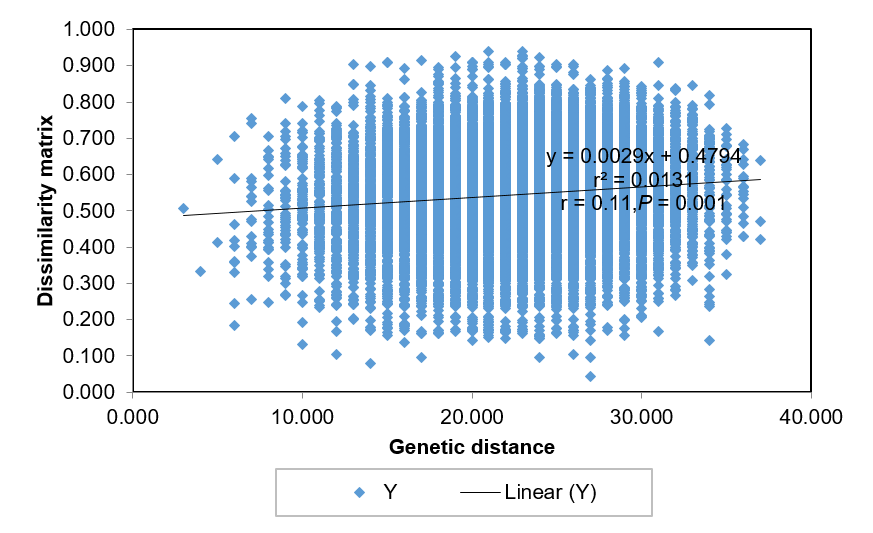

Supplement: Supplementary file 1 [file genes-12-00063-s001.zip › Supplementary files/Figuse S4.png]
